# Effect of different physical activity training methods on epilepsy: A protocol for systematic review and meta-analysis

**DOI:** 10.1097/MD.0000000000029085

**Published:** 2022-03-18

**Authors:** Chen Qi Zhang, Hong Yan Li, Yong Wan, Xue Yang Bai, Lu Gan, Hong Bin Sun

**Affiliations:** ^a^ *Department of Special-needed Medical, Chengdu BOE Hospital, Chengdu, Sichuan Province, China,* ^b^ *Department of Neurology, Sichuan Academy of Medical Sciences and Sichuan Provincial People’s Hospital, Chengdu, China.*

**Keywords:** epilepsy, meta-analysis, physical exercise

## Abstract

**Background:**

Over the past few decades, people with epilepsy were advised not to engage in sports based on the fear that sporting activity may cause injuries, potentially induce seizures, and have a negative effect on disease course. However, in recent years, numerous studies have indicated a positive role of physical exercise in reducing the frequency of seizures and improving health condition of patients with epilepsy. The purpose of this study was to compare the effects of different physical exercise programs on the symptomatology and health condition of individuals with epilepsy and provide guidance for selecting the optimal modality of physical exercise for patients with epilepsy via a meta-analysis of available literature.

**Methods:**

A literature search was carried out in MEDLINE via PubMed, Cochrane Library, EBSCO, Embase, China National Knowledge (CNKI), and Wan fang databases to gather relevant data about different physical exercise modalities and epilepsy. We will use Endnote X9 software for studies selection and Stata16.0 software for the data analysis.

**Results:**

This present study aimed to provide the most up to date evidence and recommendations for clinicians and epilepsy patients to choose an optimal type of exercise.

**Conclusion:**

Aerobic exercise and resistance exercises both had a positive effect on epilepsy patients. Persons with epilepsy should be encouraged to participate in sports activities.

INPLASY registration number: INPLASY202220070.

## 1. Introduction

Epilepsy is a chronic disease of the central nervous system that affects individuals of all ages and has a worldwide distribution. As reported by the Lancet Neurology in 2019, there are currently 46 million patients with all-active patients with epilepsy (PWE) (both idiopathic and secondary epilepsy) globally. Prevalence of active epilepsy increased with age, with peaks at 5 to 9 years and at older than 80years of age.^[1]^ Pharmacotherapy with antiepileptic drugs (AEDs) remains the major treatment modality for epilepsy. Approximately 30% of PWE continue to have seizures despite AEDs treatment.^[2]^ However, long-term use of these drugs may cause a variety of side effects and the most commonly reported adverse events across all AEDs were drowsiness/fatigue, headache or migraine, gastrointestinal disturbances, dizziness/faintness, and rash or skin disorders.^[3]^

PWE also experience a high degree of comorbidity. Physical and mental health comorbidity was more common with epilepsy, approximately 69.9% of individuals with epilepsy had 1 or more comorbid health conditions and 18.6% had 4 or more, depression was present in 16.3% of people with epilepsy compared to 9.5% of those without and this figure is continuously rising.^[4]^ Meanwhile, these comorbidities drive additional polypharmacy.^[5]^ A nationally representative sample study found that patients treated for epilepsy took an average 5.3 medications, which not only increase the medication burden but also the potential risk for adverse effects from polypharmacy.^[6]^

Physical exercise is presented as a self-management strategy, can assist in reducing the frequency of seizures, higher self-esteem and social interaction, improving long-term physical well-being and optimize mental, and reducing the disease burden for people living with epilepsy.^[7]^ However, for many years, people with epilepsy were advised not to engage in physical activity trainings. Barriers prevent many PWE from exercising safely and confidently included a fear of injury, lack of social support, stigma, over protection, exercise-induced seizures (e.g., through overheating and/or high exercise intensity level) and have a negative effect on disease processing.^[8,9]^ In addition, PWE tend to have sedentary lifestyles which may predispose them to a lower perceived quality of life.^[10]^ In particular, due to the necessary social isolation, institutional and home quarantine during the COVID-19 pandemic currently, a high proportion of PWE had a worsening of their seizure control and the reason for this seizure exacerbation was not because of COVID-19 infections but rather to other factors such as physical activity, diet, sedentary behavior, and emotional distress due to home and social confinement.^[11]^

Promoting physical activity and adequate sleep may minimize the potential impact of the pandemic in PWE.^[11,12]^ Numerous studies have shown that physical exercise has been shown to be a physiological and psychological benefit for PWE.^[9]^ However, there are still many gaps in research concerning the effect of different physical exercise modalities (including aerobic exercise, resistance exercises, stretching exercises or combined aerobic, and resistance exercises) on the symptomatology, body composition, physical activity, physical fitness, cognitive function, mental emotion (depression and anxiety), sleep quality, and quality of life of individuals with epilepsy. In the present study, we aim to systematically compare the effectiveness of different types of physical exercise in PWE and provide guidance for selecting the optimal modality of physical exercise for PWE via a meta-analysis of available literature.

## 2. Methods

### 2.1. Study registration

This study was registered on the International Platform of Registered Systematic Review and Meta-analysis Protocols with the registration number of INPLASY202220070 and the DOI number is 10.37766/inplasy2022.2.0070.

### 2.2. Selection criteria

#### 2.2.1. Participants.

The criteria for inclusion were: age > 12 years to ensure that physical exercises programs can be done accurately; patients’ diagnosis of epilepsy based on International League Against Epilepsy criteria (2017) irrespective of their seizure type or epilepsy syndrome. There is no restriction on gender, race, treatment time, region of the enrolled patients, type and number of AEDs prescribed, and seizure control.

#### 2.2.2. Interventions and comparators.

The control group will be defined as patients which did not perform any regular physical exercise during the period and just maintenance of usual activities. The intervention group will be assigned with a supervised physical exercise program.

#### 2.2.3. Outcomes.

The primary outcome measures were improvements of seizure frequency which were defined as any reduction in seizure frequency after implementation of the physical exercise; the secondary outcome indicators were health variables based on comorbidities of pediatric and adult epilepsy (e.g., depression, anxiety, psychiatric symptoms, sleep quality, overall quality of life) and changes in body composition. The incidences of adverse events associated to physical exercise will also be included as secondary outcomes.

#### 2.2.4. Study design.

To decrease the risk of bias in individual studies, only randomized controlled blind/double-blind studies which reported the interest outcomes were included in this metaanalysis. We excluded: non-randomized controlled trials study designs, for example non-randomized concurrent control trials, before-after studies, and cohort studies; we excluded animal studies, commentaries, editorials, conference abstracts, metaanalysis, and letters without sufficient data; and unclearly defined epilepsy.

### 2.3. Search strategy

In order to search all relevant literatures, electronic and manual searching were combined. We searched the following electronic databases: MEDLINE via PubMed, Cochrane Library, EBSCO, Embase, China National Knowledge (CNKI), and Wan fang databases (up to February 2022). We will conduct by using medical subject headings (Mesh) and term words, such as “Exercises[Mesh]”, “Sports[Mesh]”, “ Physical Fitness[Mesh]”, “raining*”, “physical Activit*”, “Epilepsy[Mesh]”, “epileps*”, “seizure*”, “Convulsion*”, and so on. Additionally, we also searched the bibliography of retrieved articles to obtain relevant articles.

### 2.4. Study selection and data extraction

We will use Endnote X9 software (Captivate Analytics, USA, version EndNote X9.3.2) for document screening and document management. The titles and abstracts of all studies derived from the search results were read thoroughly to confirm eligibility by 2 researchers independently (ZCQ and LHY), and the full-text versions of all potentially eligible studies were then retrieved for further assessment. Any discrepancies or divergences were resolved through discussion and consensus, and when in doubt, the final decision was made in consultation with a third author (WY). The data extracted from the included studies are including: the first author’s name, publication date, rational, sample sizes, mean age, gender, details of participants (e.g., diagnostic criteria, epilepsy types or syndrome, AEDs, etc), interventions (e.g., means and duration times of physical exercise), interested outcomes, and adverse events.

### 2.5. Quality assessment

The quality of each article was appraised by 2 individual authors (ZCQ and BXY) using the Cochrane Risk of Bias assessment tool^[13]^ for randomized controlled trials. The domains including the following 7 aspects, include random sequence generation, allocation concealment, blinding of the participants, blinding of outcome assessments, incomplete outcome data, and selective outcome reporting.

### 2.6. Statistical analysis

Stata 16.0 (Stata Corporation, College Station, TX) software will be performed for statistical analyses. Dichotomous variables are expressed as risk ratios and the precision of the estimates is evaluated by the 95% confidence interval. The Q test is used to assess the presence of heterogeneity, and the I^2^ index is used to quantify the extent of heterogeneity. If I^2^ < 50%, the heterogeneity is small, data will be analyzed using a fixed-effects model. If I^2^ > 50% and *P* < .05, data will be analyzed using a random effects model. Standardized mean difference with the 95% confidence interval as an effect size is measured for continuous data. Had we had sufficient data, we would have carried out the subgroup analyses (e.g., duration times of physical exercise and types of physical exercise). In addition, we will conduct sensitivity analyses to assess the robustness of the results and carry out an assessment of publication bias using funnel plots to assess the risk of reporting bias.

### 2.7. Confidence in cumulative evidence

The strength of evidence will be assessed using the Grading of Recommendations Assessment, the domains including the following 5 aspects (risk of bias, indirectness, inconsistency, imprecision, and publication bias) which be divided into 4 levels: very low, low, moderate, and high.^[14,15]^ This Grading of Recommendations Assessment system will help to define the resulting evidence of the systematic review.

### 2.8. Ethics and dissemination

Ethics and dissemination ethical approval are not required since it is secondary data analysis based on published literature.

## 3. Discussion

Evidence from animal studies has indicated that chronic cardiopulmonary exercise (voluntary wheel running or forced swimming)^[16]^ and resistance exercise programs^[17]^ both had beneficial effects on controlling seizure through enhancement of cognition and highlights the possibility to translate into reduced seizure occurrence in PWE. The positive actions of physical exercise on the brain were also demonstrated in clinical observational studies. A retrospective cohort study in Swedish cross-country skiers showed that faster skiers revealed lower incidence of epilepsy compared to slower skiers.^[18]^ Aerobic dancing exercise (combine strength training and stretching) for 60 minutes can significantly reduce the self-reported seizure frequency and the level of subjective health complaints, such as muscle pains, sleep problems, and fatigue.^[19]^ According to Häfele et al,^[20]^ a 60-minute structured physical exercise program including warm-up (5-minutes), aerobic training (15-25 minutes at 14-17 on Borg scale), resistance training (2-3 sets, 10-15 repetitions), and stretching improved overall health of PWE and decreased seizure frequency.

Although numerous meta-analyses and studies proved that regular physical exercise had a positive consequence on the health of PWE including cognitive function, emotional lability, overall quality of life and sleep quality, and seizure frequency.^[7,9,21-23]^ However, little work has been made on how PWE chooses the physical exercise programs that best fit their overall healthy conditions and lifestyle. The optimal physical exercise modality for PWE is still an open question.

In 2016, the International League Against Epilepsy published a consensus paper that recommends safe sports participation for PWE.^[24]^ Sports are divided into 3 categories based on potential risk of injury or death should a seizure occur: sports with no significant additional risk, including bowling, judo, wrestling, collection sports on the ground, cross-country skiing, curling, dancing, golf, etc; sports with moderate risk to PWEs, but no risk to bystanders, including alpine skiing, archery, biathlon, triathlon, modern pentathlon, canoeing, boxing, cyding, fencing, ice hockey, shooting, skating, swimming, water skiing, weightlifting, etc; and sports with major risk, including aviation, climbing, diving, horse racing, motor sports, parachuting, rodeo, scuba diving, ski jumping, solitary sailing, surfing, etc. At the same time, International League Against Epilepsy still suggested that several factors should be considered when advising whether a PWE can participate in specific activities include the type of sport, including the probability of a seizure occurring, the type and severity of the seizures, seizure precipitating factors, the usual timing of seizure occurrence, and the person’s attitude in accepting some level of risk.^[24]^

Despite previous studies held objective evidence suggests that physical exercise had a clear positive effect in PWE. However, the ways of physical exercise are also diversified and the physical practice of which can vary enormously for PWE. Currently, there is no clinical research regarding the effect of different physical exercise modalities on the PWE yet. The present study was performed to compare the effectiveness of different types of physical exercise in individuals with epilepsy and aimed to provide the most up to date evidence and recommendations for clinicians and epilepsy patients to choose an optimal type of exercise.

## Author contributions

**Conceptualization:** Chen Qi Zhang, Hong Bin Sun.

**Data curation:** Chen Qi Zhang, Hong Yan Li, Xue Yang Bai.

**Formal analysis:** Chen Qi Zhang.

**Investigation:** Hong Yan Li, Hong Bin Sun, Chen Qi Zhang, Lu Gan.

**Methodology:** Chen Qi Zhang, Yong Wan, Xue Yang Bai, Hong Bin Sun.

**Software:** Chen Qi Zhang, Yong Wan.

**Supervision:** Hong Yan Li, Lu Gan, Hong Bin Sun.

**Validation:** Hong Yan Li, Lu Gan, Hong Bin Sun.

**Writing** - **original draft:** Chen Qi Zhang.
